# The Rise of *Coccidioides*: Forces Against the Dust Devil Unleashed

**DOI:** 10.3389/fimmu.2019.02188

**Published:** 2019-09-11

**Authors:** Marley C. Caballero Van Dyke, George R. Thompson, John N. Galgiani, Bridget M. Barker

**Affiliations:** ^1^Pathogen and Microbiome Institute, Northern Arizona University, Flagstaff, AZ, United States; ^2^Department of Medical Microbiology and Immunology, University of California, Davis, Davis, CA, United States; ^3^Division of Infectious Diseases, Department of Internal Medicine, University of California Davis Medical Center, Sacramento, CA, United States; ^4^Valley Fever Center for Excellence, Department of Medicine, University of Arizona College of Medicine-Tucson, Tucson, AZ, United States

**Keywords:** *Coccidioides*, valley fever, fungal vaccines, antifungal drugs, immunity

## Abstract

Coccidioidomycosis (Valley fever) is a fungal disease caused by the inhalation of *Coccidioides posadasii* or *C. immitis*. This neglected disease occurs in the desert areas of the western United States, most notably in California and Arizona, where infections continue to rise. Clinically, coccidioidomycosis ranges from asymptomatic to severe pulmonary disease and can disseminate to the brain, skin, bones, and elsewhere. New estimates suggest as many as 350,000 new cases of coccidioidomycosis occur in the United States each year. Thus, there is an urgent need for the development of a vaccine and new therapeutic drugs against *Coccidioides* infection. In this review, we discuss the battle against *Coccidioides* including the development of potential vaccines, the quest for new therapeutic drugs, and our current understanding of the protective host immune response to *Coccidioides* infection.

## Introduction

**Figure 1:**
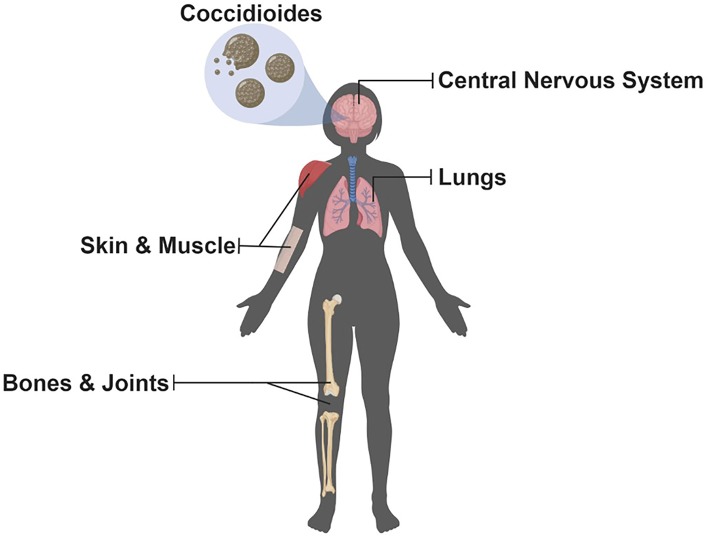
Potential Organs Infected by *Coccidioides*. Since inhalation is the most common route of infection, the lungs are the most common organ that becomes infected with *Coccidioides*; however, dissemination can occur allowing for multiple organs, highlighted above, to become infected but are uncommon (Illustration created with BioRender).

The *Coccidioides* genus contains *C. immitis* and *C. posadasii*, the etiological agents of Valley fever. This neglected disease occurs primarily in the southwestern United States, most notably in California and Arizona; however, cases have appeared in Washington pointing to an underappreciation of the geographic distribution of this organism (1, 2). Furthermore, cases outside the United States have been occurring in the northern region of Mexico (3) and areas of Central and South America (4, 5). *Coccidioides* is considered both a primary and opportunistic fungal pathogen occurring in both immunocompetent and immunocompromised individuals causing a spectrum of coccidioidomycosis. Most cases (~60%) are asymptomatic. For the remainder, pulmonary symptoms from underlying acute or progressive pneumonia are the most common reason patients seek medical help (6, 7). Additionally, dissemination can occur affecting a multitude of organs (Figure 1) and lead to the most severe complication, coccidioidal meningitis. Originally, the literature stated that an estimated 150,000 infections occur each year in the United States, and about 1% lead to disseminated disease with a third of those being fatal (7). Host factors strongly influence risk of disseminated disease such as immunosuppression, third trimester of pregnancy, old age, and ethnicity (i.e., African Americans and Filipinos) (6–12). Additionally, host response to treatment varies, current antifungals cause potential adverse side effects, and resistance to antifungals has recently become a concern (13, 14). Furthermore, infections caused by *Coccidioides* are on the rise (15), and new estimates of the annual number of new U.S. infections are more than twice (350,000) that of previous estimates (16). The reason for the increase in *Coccidioides* cases is largely unknown; however, factors such as changes in the environment and surveillance methodology could be contributing factors (15). Taken together, there is an urgent need for new antifungal agents, a better of understanding of host response to infection, and the development of a vaccine to combat coccidioidomycosis. Here, we review the current understanding of the host immune response to infection and protection, advances in drug development, and discuss promising approaches to developing a *Coccidioides* vaccine; a one stop-shop to understand current research in the battle against the Dust Devil.

## Host Immune Response to *Coccidioides*

*Coccidioides* grow in the soil as fungal mycelia which segment into arthroconidia (spores) that can then become aerosolized, inhaled, and cause infection. Once a host is infected, arthroconidia transition into mature rupturing spherules within 5 days of infection (17). Therefore, during the early days of infection, morphological variation of *Coccidioides* is high as the organism is switching from arthroconidia to its parasitic stage, the spherule. In this section, we will discuss what is known about the host immune response to *Coccidioides* infection, first focusing on the early immune response, and then discussing the protective host immune response to battle coccidioidomycosis.

### Early Innate Immune Response to *Coccidioides*

The innate immune response is the first line of defense against fungal pathogens and clearance relies heavily on phagocytosis by macrophages and neutrophils (18). Phagocytosis can occur on inhaled arthroconidia (3–5 μm) (17) and endospores (2–7 μm); however, mature spherules are too large (15–80 μm) (19) and phagocytic cells fail to engulf these fungal organisms (20). Neutrophils for example can only partially engulf cells that are about 11 μm (21) which is below the threshold of the size of a mature spherule. Studies have shown an influx of neutrophils during infection with *Coccidioides* (22) and when spherules burst releasing hundreds of endospores (23, 24). Past reports have shown C57BL/6 mice depleted of neutrophils are as susceptible as wild-type mice when infected with wild type *Coccidioides* (19). Conversely, when mice are vaccinated with a live-attenuated strain of *Coccidioides* (ΔT, genetically engineered mutant originally designated Δ*cts2/ard1/cts3*), protection relies on the presence of neutrophils (19). Additionally, studies conducted by Gonzalez et al. showed that mice deficient in NADPH oxidase (NOX2) were more susceptible to infection with *C. posadasii* compared to wild type mice while inducible nitric oxide synthase (iNOS) knock-out mice demonstrated that iNOS does not play a significant role in the control of *Coccidioides* infection (25, 26). Interestingly, NOX2^−^/^−^ mice had substantially more infiltration of neutrophils in the lungs compared to wild type mice while iNOS^−^/^−^ mice had a significant increase of neutrophils at day 7 but not day 11 post challenge. Overall these studies demonstrate that neutrophils play a role in the proper inflammatory response during a *Coccidioides* infection, and dysregulation of an inflammatory response can be detrimental to the host.

*Coccidioides* spherules can also escape phagocytosis from macrophages (23). Macrophages vary in size depending on location in the host: 5 μm spleen, 10 μm peritoneal surface, and 15 μm alveoli (27). Studies have demonstrated an evolutionary conserved particle/pathogen size ratio contributes to pathogen clearance and recognition (28), suggesting the inability of macrophages to phagocytose mature spherules. Vaccination studies have demonstrated the influx of macrophages to the lungs of vaccinated mice compared to unvaccinated mice after challenge with *Coccidioides* (22). However, the role of macrophage subsets (i.e., classically and alternatively activated macrophages or M1 and M2 macrophages) in the protective host immune response against *Coccidioides* has yet to be elucidated. Studies have shown that mouse peritoneal macrophages stimulated with *Coccidioides* spherules produce tumor necrosis factor alpha (TNF-α) (29). Furthermore, studies have shown increases in cytokines such as interferon gamma (IFNγ), tumor necrosis factor alpha (TNFα), and interleukin (IL)-17 in mononuclear cells from bronchoalveolar lavage fluid (BALF) from patients with pulmonary coccidioidomycosis (30).

Studies further determined which pattern recognition receptors (PRRs) on peritoneal macrophages were important for recognition of *C. posadasii* spherules. Using peritoneal macrophages from wild-type cells compared to different knockout mice (i.e., TLR2^−^/^−^ and MyD88^−^/^−^), results demonstrate the response to spherules is dependent on Toll-like receptor 2 (TRL2), myeloid differentiation factor 88 (MyD88), and Dectin-1 (31). Dectin-1 is a C-type lectin receptor shown to interact with components of the fungal cell wall. Studies have shown the importance of this C-type lectin receptor where Dectin1^−^/^−^ mice infected with *Coccidioides* demonstrated increased pulmonary fungal burden and decreased Th17 cytokines (32). Studies further suggest that increased susceptibility of C57BL/6 mice to coccidioidomycosis is due to alternative splicing of the Dectin-1 gene (33). Furthermore, studies have identified that null mutations in Dectin-1 predispose hosts to chronic mucosal candidiasis (34). Additionally, people with mutations in the *CARD9* gene, Dectin-1, and other C-type lectin receptors signaling through this gene have increased susceptibility to fungal infections (35). Another C-type lectin receptor, the mannose receptor, has been shown to be important in the immune response of human coccidioidomycosis but does not play a role in a murine model of coccidioidomycosis (36–38). Studies demonstrated an association with low mannose-binding lectin (MBL), a collectin that is part of the innate immune system, serum levels among patients exhibiting an active *Coccidioides* infection compared to otherwise healthy individuals; however, the role of MBL in the pathogenesis of *Coccidioides* has yet to be determined (37). Recent studies further investigated the role of multiple receptors that use MyD88 to determine which of these receptors are required for resistance against coccidioidomycosis. Of all the surface receptors investigated, results from the studies determined IL-1R1 signaling to be important for protection against coccidioidomycosis (39). Overall, these studies demonstrate the potentially crucial role of C-type lectin receptors and certain TLRs to protect against coccidioidomycosis, but much remains to be done.

Dendritic cells (DCs) act as a bridge between the innate and adaptive immune response. DCs initiate the immune response by capturing antigens and then activate and modulate lymphocytes. Mature DCs have the ability prime naïve T cells toward phenotypes (Th1 and Th17) protective against coccidioidomycosis (discussed below) (40). Studies have demonstrated that DCs pulsed with *Coccidioides* antigen (spherulin, spherule lysate) can activate DC maturation and lymphocyte proliferation in non-immune individual cells (41). Furthermore, studies investigated the effects of DCs pulsed with a coccidioidal antigen preparation, T27K, using PBMCs from patients with disseminated coccidioidomycosis compared to healthy individuals (42). Results from these studies demonstrate that DCs can be generated by patients with disseminated coccidioidomycosis, and stimulation with T27K led to increased IFN-γ levels in both disseminated and healthy patient samples. Furthermore, studies have demonstrated that suppressing DC responses led to defective T cell responses. BALB/c mice are highly susceptible to infection with *Coccidioides*, whereas DBA/2 mice are more resistant. Bone-marrow derived DCs (BMDCs) from DBA/2 mice infected with *Coccidioides* demonstrated an increase in IL-12 secretion and T cell co-stimulatory cell surface molecules compared to BALB/c mice (43). Thus, these studies suggest BALB/c mice could be more susceptible due to impaired DC responses; however, more studies are needed using other mouse strains that are susceptible to infection with *Coccidioides*.

Despite species divergence of *C. immitis* and *C. posadasii* about 5.1 million years ago (44), many studies state that these two species cause similar disease clinically. However, studies from our laboratory allude to differential early host innate responses among species of *Coccidioides* in a murine model of coccidioidomycosis (45). Since host responses strongly influence clinical disease, differences in the first line of defense against coccidioidomycosis could attribute to differences in outcome of disease. Mice were infected with 1 × 10^5^ arthroconidia of either a *C. immitis* pure strain (2006), *C. immitis* hybrid strain (RS), or a *C. posadasii* pure strain (Silveira). Real-time RT-PCR analysis of mouse lungs shows differential responses across strains. Expression of proinflammatory cytokine levels (IL-1α and IL-17α) were significantly increased in the mice infected with the 2006 strain (*C. immitis*) at day 5 post infection compared to all other infected mice. Silveira (*C. posadasii*) infected mice demonstrated an increase in proinflammatory cytokine IL-1β at day 1 post infection and immunoregulatory cytokine IL-10 at day 5 post infection compared to other strains (45).

*Coccidioides* has other means of avoiding phagocytosis and evading the immune response. Spherule outer wall glycoprotein (SOWgp) is a major antigen present on the cell surface of *Coccidioides* (46, 47). This glycoprotein is highly expressed during the transition to spherules, and demonstrates immunogenic properties (46, 47). Interestingly, studies have shown that a specific metalloproteinase (Mep1) is secreted during endosporulation, which then digests SOWgp to prevent host recognition (48). Furthermore, mice vaccinated with recombinant SOWgp and then challenged with a *C. posadasii* strain with the *MEP1* gene disrupted demonstrated increased survival compared to the parental or revertant strain (48). Other studies have demonstrated that *Coccidioides* can suppress nitric oxide (NO) production in macrophages; however, these studies show NO is not critical for *in vitro* killing of *Coccidioides* (49). Although these studies give us insights into *Coccidioides* pathogenesis, more studies are needed to understand the immune evasion strategies of this pathogen.

### Protective T-Cell Host Immune Response to Combat *Coccidioides*

Results from both clinical data and mouse models of coccidioidomycosis have demonstrated that T cell immunity is crucial for protection against coccidioidomycosis. Additionally, deficiency in CD4^+^ T cells results in increased susceptibility to infection with *Coccidioides* (50). CD4^+^ T cells can differentiate into distinct lineages that produce certain cytokines in response to a pathogen. Cytokines such as IL-12 and IFN-γ are associated with T cell helper 1 (Th1) responses, which has been shown to be important for protection in mouse models of coccidioidomycosis (51, 52) and *in vitro* studies using human PBMCs (53). Additionally, patients with IL-12 and IL-1 receptor deficiencies demonstrate increased dissemination of *Coccidioides* (54, 55). A Th2 immune response is activated by cytokines such as IL-4 and IL-5 and has been shown to downregulate the host immune response during infection with *Coccidioides* (51). On the other hand, these cytokines can induce B cell responses which have been shown to play a role in protection in a mouse model of coccidioidomycosis (51, 56, 57). However, the role of Th2 and antibodies in the clearance of *Coccidioides* has yet to be resolved and requires further study. Additionally, the detection of anti-*Coccidioides* antibodies for the diagnosis of coccidioidomycosis is not reliable in humans (58). Recently, the role of Th17 responses which produce proinflammatory cytokines such as IL-17 and IL-22 has been investigated (59). Vaccination studies by Hung et al. demonstrate the critical role of Th17 responses in protection against coccidioidomycosis (22). In these studies, mice lacking the IL-17 receptor that were vaccinated with the ΔT strain were highly susceptible to challenge with *Coccidioides*. Furthermore, mice deficient in IFN-γ and IL-4 receptors were still protected against challenge with *Coccidioides* equivalent to wild-type mice. Thus, demonstrating conflicting results of the importance of IFN-γ in the protection against coccidioidomycosis. These studies also demonstrate the immune response of ΔT vaccinated mice challenged with *Coccidioides* is a mixed Th1, Th2, and Th17 response (22). Overall, studies demonstrate that each of these subsets play a role in the protection against coccidioidomycosis.

Along with CD4^+^ T cells, mouse studies show that CD8^+^ T cells play a role in protection against infection with *Coccidioides* (60). Studies have shown an increased percentage of CD8^+^ T cells were present post challenge among ΔT vaccinated mice compared to non-vaccinated mice (22). Importantly, BALF from patients with coccidioidomycosis demonstrated an increased proportion of CD8^+^ T cells in patients with acute pulmonary *Coccidioides* infection compared to all other groups (30). Additionally, studies have shown that CD8^+^ T cells can compensate for the lack of CD4^+^ T cells and confer protection against fungal pathogens (60–63). Studies analyzing pediatric patients with coccidioidomycosis demonstrated an overall lower adaptive immune response in persistent disease patients with a trend toward lower CD4^+^ and CD8^+^ T cells, and significantly fewer B cells compared to control and resolved patients (64). Additionally, these studies found no difference in Th1 frequencies among patient populations; however, patients with persistent disease had a lower frequency of Th17 and higher T regulatory (Treg) frequencies compared to patients with resolved disease. Therefore, studies from both human and mouse models of coccidioidomycosis have demonstrated an association between increased Th17 responses and resolution of infection.

## Development of a *Coccidioides* Vaccine

Despite earnest efforts, there is currently no clinically available vaccine against any fungal organism; although, early results have been favorable in the development of a *Candida* vaccine (65). The overall goal of an anti-coccidioidal vaccine is to prevent disease. Immunization against coccidioidomycosis appears possible since patients who have recovered from an initial coccidioidal infection rarely become ill from a second infection and additional exposure (66). The first experimental anti-*Coccidioides* vaccine developed was the formalin-killed spherule (FKS) vaccine that demonstrated promising results in mice (67). However, human trails established no differences between FKS-vaccinated group and the placebo group (68). Additionally, the FKS-vaccinated group experienced severe side effects at the local injection site. Herein, we discuss various strategies to develop a vaccine to combat coccidioidomycosis.

### Live Attenuated Vaccines

Live attenuated strains have proven to be successful in stimulating the immune response similar to a naturally occurring infection (69–72). However, an ideal vaccine candidate needs to have an impeccable safety profile in all populations such as the immunocompromised (73). Although a live vaccine may not be useful in a human clinical setting, understanding the protective host immune response against *Coccidioides* is imperative to design a suitable and effective recombinant vaccine to combat coccidioidomycosis. For example, chitinase activity in *C. posadasii* was inhibited by disrupting two chitinase genes (*CTS2* and *CTS3*) and a third gene contiguous to *CTS3*, to obtain an attenuated mutant that was no longer able to endosporulate, Δ*cts2/ard1/cts3* (51). This genetically engineered strain demonstrated protection in mice against coccidioidomycosis and is now designated as the ΔT vaccine strain (51, 74). Using this vaccine strain, studies have demonstrated the important parameters for eliciting a protective host immune response against coccidioidomycosis. As discussed above, the ΔT vaccine helped to elucidate the important role of CD4^+^ T cells, particularly Th1 and Th17 immune responses that are critical for protection (22).

More recently, a homolog of the gene *CPS1*, a virulence factor found in a maize pathogen (75), was deleted in a strain of *C. posadasii* (76). This deletion resulted in essentially complete attenuation of pathogenicity in both wild type and immunodeficient mice. Furthermore, mice vaccinated with live ΔCPS1 were protected against an otherwise lethal infection with wild type *C. posadasii* and *C. immitis* (76, 77). Further studies demonstrated a primarily Th1-type response in mice vaccinated with ΔCPS1 and challenged with wild-type *C. posadasii* compared to unvaccinated mice (77). Both the ΔT and ΔCPS1 strains are vital tools needed to determine the protective host immune response needed to battle *Coccidioides*. Interestingly, both of these mutant strains undergo initial spherulation in the host before arresting growth.

A practical attraction of a live attenuated *Coccidioides* vaccine candidate is that manufacturing costs to make a clinically feasible product should be low. Production costs have been a road block for an earlier recombinant vaccine (78); however, as with any live vaccine, safety is a critical consideration. Since ΔCPS1 is a complete gene-deletion, reversion is hard to imagine. On the other hand, new mutations in other genes might compensate for the missing gene and result in gain-of-function and cause disease, especially in more immunosuppressed individuals (73, 79). ΔCPS1 is currently being developed as a live vaccine candidate to prevent Valley fever in dogs (80). Should this prove successful, it would provide a proof-of-concept supporting further development to prevent Valley fever in humans. The exact path for this vaccine candidate to humans has yet to be determined. There is no precedent since a live attenuated eukaryotic vaccine has yet to be given FDA approval. Furthermore, the market for a vaccine to prevent Valley fever is relatively small. While there is a very strong public case for preventing this disease (81), it is much more challenging to make a business model with a return on investment competitive with other opportunities for investors. It is likely that a Valley fever vaccine will only be developed if public resources, state or federal, are deemed appropriate for this purpose.

### Novel Adjuvants and Protein Vaccines

A safer alternative to attenuated vaccines is the use of recombinant proteins; yet, these may require an adjuvant to strengthen the immune response and optimize efficacy (82). Studies sought to characterize a novel adjuvant, a peptide agonist of the biologically active C-terminal region of human complement C5a referred to as EP67, conjugated to the live ΔT vaccine strain (83). These studies found that BALB/c mice immunized with the EP67-conjugated vaccine demonstrated increased survival rates and reduced fungal burden compared to the non-conjugated vaccine. Additionally, mice given the conjugated vaccine had increased infiltration of macrophages and DCs by day 7 post challenge while neutrophil numbers were decreased by 11 days post challenge compared to the non-conjugated vaccinated mice. Furthermore, the novel adjuvant EP67 increased Th1 and Th17 immune responses; therefore, augmenting T cell immunity and enhancing protective efficacy of the live ΔT vaccine strain (83).

Early studies suggest multivalent vaccines are more effective against coccidioidomycosis compared to a single peptide vaccine (84–86). Early studies introduced rAg2/Pra as a potential vaccine candidate; however, varying routes of challenge led to conflicting results (87, 88). Thus, improved protection efficacy against *Coccidioides* infection in mice by adding an *Coccidioides*-specific antigen (CSA) to the rAg2/Pra were completed (84). The inclusion of another antigen Prp2, and development of a combined vaccination of rAg2/Pra+rPrp2, produced significantly improved protection compared to either of the recombinant proteins alone (89). Additionally, recent studies have demonstrated a Ag2/Pra-specific response in mice using a DC-based vaccine which was prepared by transfecting primary bone marrow-derived DCs with a plasmid encoding Ag2/Pra (90). Prior studies demonstrated that the DC-based vaccine reduced fungal burden and increased IFNγ levels in the lung homogeneates from vaccinated mice compared to control mice (91).

Using two-dimensional gel electrophoresis and high-performance liquid chromatography-tandem mass spectrometry (HPLC-MS/MS), studies identified another protein, *PMP1* (peroxisomal matrix protein 1), which also demonstrated protection in a mouse model of coccidioidomycosis (92). Additional protective antigens that were used as potential vaccine candidates include *PEP1, PLB*, and *AMN1*, which demonstrated enhanced protection as a multivalent vaccine compared to a single antigen alone (85). An alternative approach to a multivalent vaccine to lower cost is the used of epitope-based vaccines (EBV) which has been shown to effectively induce an immune response, is relatively easy to produce, and expected to be safe to use in humans (93, 94). Studies conducted by Hurtgen et al. created a recombinant EBV (rEBV) which incorporated *PEP1, AMN1*, and *PLB* into a single epitope-based vaccine which was either admixed with an adjuvant or loaded into glucan particles (GPs) (95). Overall, these studies demonstrated that the rEBV plus GP vaccination was superior to all formulations tested in this study showing enhanced survival, reduced fungal burden, and robust Th1 and Th17 immune responses compared to control mice with GPs alone. GPs are purified, hollow, porous yeast cell-wall particles derived from *Saccharomyces cerevisiae*. There have been several types of yeast particles created for vaccine development (96).

Recently, studies created a recombinant chimeric polypeptide antigen, rCPa1, that consist of Ag2/Pra, Cs-Ag, Pmp1, and 5 T cell epitopes from *PEP1, PLB*, and *AMN1* from *C. posadasii* (97). Additionally, they tested the efficacy of rCpa1 encapsulated in differently formulated yeast cell-wall particles. These studies identified a promising vaccine candidate, rCpa1, encapsulated in glucan-chitin particles (GCP-rCpa1) that showed increased survival, significantly reduced fungal burden, and a mixed protective Th1 and Th17 response (97). Additionally, recent studies conducted by Hayden et al. demonstrated that mice immunized with recombinant Ag2 expressed in maize and loaded into GCPs had reduced fungal burden in *Coccidioides* challenged mice similar to Ag2 derived from *Escherichia coli* (98). Furthermore, oral administration of Ag2 fused onto a DC carrier peptide (DCpep) demonstrated protective Th17 responses. More studies are needed to characterize these new vaccine candidates to determine if clinical trials are on the horizon. To move into clinical trials, we need to test potential vaccine candidates in multiple animal models including transgenic mice expressing human receptors.

## Current Treatments and Drug Discovery

Coccidioidomycosis represents a spectrum of illness ranging from asymptomatic acquisition with resultant immunity to severe and life-threatening disseminated infections. Even in otherwise uncomplicated primary pulmonary infection the symptoms of fever, chills, cough, joint pain, and malaise can last weeks to months (99). Severe cases including dissemination to the skin, bone, or brain (Figure 1) can be difficult to treat and in some cases require life-long antifungal therapy. Currently, the most common management of coccidioidomycosis includes antifungal agents such as fluconazole or itraconazole; however, guidelines suggest an individualized approach to patient management (13). Nevertheless, new concerns of toxicities and side effects, with either acute or long-term use, caused by these agents have seen renewed interest in the development of new agents to combat this disease. Herein, we discuss briefly the current and future treatment options for patients with coccidioidomycosis. A comprehensive review of current treatment options against coccidioidomycosis has recently been published (100).

### Polyenes

Amphotericin B has been a widely used agent in the treatment of coccidioidomycosis over the last 50 years (101) and is currently available in multiple intravenous formulations: amphotericin B deoxycholate (AmBd), liposomal amphotericin B (L-AMB), amphotericin B colloidal dispersion (ABCD), and amphotericin B lipid complex (ABLC) (Table 1) (100). Overall, these formulations are met with adverse effects such as nephrotoxicity, hypokalaemia, phlebitis, fever, chills, hepatotoxicity, and anemia (102–105). Historically, long courses of amphotericin B therapy was prescribed in an attempt to provide curative therapy given the lack of an orally available efficacious agent. With the availability of the less toxic triazole antifungals, amphotericin B therapy is reserved for the treatment of patients who are intolerant or refractory to the other available antifungal agents or those with severe disease.

**Table 1:** A brief overview of antifungal agents benefits, weaknesses, and adverse effects in the treatment of coccidioidomycosis.

**Antifungal agent**	**Benefits**	**Weaknesses**	**Adverse effects**
**Triazoles**			
Fluconazole	Low cost/tolerable	High MIC values *in vitro*	Hepatotoxicity, QTc prolongation, alopecia, xerosis, and cheilitis
Itraconazole	Highly efficacious and tolerable	CSF and bone penetration, TDM	Hepatotoxicity, gastrointestinal distress, hypertension, hypokalemia, negative inotrope, and peripheral edema
Voriconazole	High CSF penetration	Variable bioavailability and TDM	Hepatotoxicity, photopsia, and photoxic skin reactions, visual hallucinations, rashes/long-term use lead to skin carcinoma, alopecia, and xerosis
Posaconazole	Penetrates most body sites and effective against nonmeningeal coccidioidomycosis	Therapeutic drug monitoring advised, low/variable CSF penetration	Gastrointestinal distress, hypokalemia, hypertension, peripheral edema
Isavuconazole	Efficacious against primary coccidioidomycosis, prolonged half-life, and tolerable	Limited clinical data against meningeal coccidioidomycosis	Gastrointestinal distress and hypokalemia
**Polyenes-Amphotericin B**			
AmBd	Intrathecal route	Highly toxic	Nephrotoxicity, hepatotoxicity, hypokalemia, phlebitis, fever, chills, dyspnea, chest/back pain
ABCD	N/A		
ABLC	N/A		
L-AMB	Less renal toxicity		

*AmBd, amphotericin B deoxycholate; ABCD, amphotericin B colloidal dispersion; ABLC, amphotericin B lipid complex; L-AMB, liposomal amphotericin B*.

There have been numerous studies using animal models that have demonstrated the efficacy of the lipid formulations of amphotericin B therapy against coccidioidomycosis (106–109). Although clinical studies have been sparse, the use of amphotericin B against multiple forms of coccidioidomycosis has demonstrated its efficacy. A retrospective study demonstrated similar efficacy of ABLC and L-AMB in the treatment of severe coccidioidomycosis; however, L-AMB may be the preferred agent with less renal toxicity during treatment compared to ABLC (110). Studies have shown that coccidioidal meningitis treated with amphotericin B deoxycholate via the intrathecal route demonstrates a much more successful treatment compared to the intravenous route (111). However, discussion with those experienced in the treatment of intrathecal therapy is highly recommended if intrathecal therapy is needed during clinical care given the potential morbidity with treatment via this approach (112, 113).

### Triazoles

The triazoles used to combat coccidioidomycosis include: fluconazole, itraconazole, voriconazole, posaconazole, and isavuconazole. These triazoles prevent the conversion of lanosterol to ergosterol thus affecting ergosterol synthesis. More specifically, these agents, with significant affinity differences, inhibit cytochrome P450 (CYP)-dependent 14-α-demethylase (114). This affinity difference leads to variability among the antifungal agents in their efficacy, spectrum of activity, and side effect profile. Despite the commercial availability of the triazoles, few have been evaluated in prospective clinical trials due to the regional nature of the disease and the high financial burden of these types of studies. However, the designation of coccidioidomycosis as an orphan disease may facilitate these efforts and allow future antifungal agents to be fully evaluated in prospective fashion.

#### Fluconazole

Fluconazole is the most frequently prescribed antifungal agent and clinical guidelines suggest it to be a first line agent against multiple forms of coccidioidomycosis (13). Advantages of this agent include low cost, tolerability, the availability of both an oral and intravenous formulations, long half-life, and excellent bioavailability (see Table 1 for an overview of benefits, weaknesses, and adverse effects) [for pharmacokinetics of antifungal agents see recent review article (100)]. Fluconazole has the ability to penetrate most tissues with adequate concentrations within the cerebrospinal (CSF) fluid allowing for the treatment of coccidioidal meningitis (CM) (13, 115). Although adverse effects from the use of fluconazole are largely benign, patients have experienced hepatotoxicity, heart corrected QT interval prolongation, alopecia, xerosis, and cheilitis (100, 116).

A recent study performed a large-scale susceptibility test to understand triazole minimum inhibitory concentrations (MICs) of *Coccidioides* isolates. These results revealed increased fluconazole MICs across multiple *Coccidioides* isolates tested (≥16 μg/ml, 37.3% of isolates; ≥32 μg/ml, 7.9% of isolates) (14). This decreased *in vitro* susceptibility of fluconazole may explain the need for higher fluconazole doses during treatment of coccidioidomycosis (13) and a dose-dependent response to fluconazole has been observed using a murine model of systemic coccidioidomycosis (117); however, this *in vitro* data has yet to be correlated with clinical outcomes. At this time, no comparative trial has evaluated the dose-dependent response of fluconazole in a randomized study; although, efficacy has been definitively demonstrated (118). Recently, tolerability of long-term fluconazole therapy was assessed, and it was demonstrated that out of 124 patients ~50% had adverse effects (116). The most common adverse effects patients experienced included xerosis, alopecia, and fatigue, which resulted in 65% of patients requiring a therapeutic change.

#### Itraconazole

Itraconazole is also frequently prescribed to treat coccidioidomycosis (13). This antifungal agent is available primarily as a capsule or oral solution (100). Advantages of using itraconazole include long half-life, efficacy, and tolerability (Table 1), although gastrointestinal side-effects are common with the oral solution, negative inotropic effects on cardiac output have been reported (119). Also, recent reports describe the development of hypertension following itraconazole initiation (120). However, the bioavailability is highly variable and studies have shown itraconazole to exhibit poor CSF (121, 122) and bone penetration (123). Additionally, due to variable bioavailability, therapeutic drug monitoring is recommended to ensure adequate absorption (124).

Despite poor CSF and bone penetration, studies have shown itraconazole to be highly efficacious in the treatment of both osseous coccidioidomycosis and coccidioidal meningitis (118, 125–127). Galgiani et al. compared fluconazole and itraconazole therapy in non-meningeal coccidioidal infections. These studies demonstrated an enhanced response in itraconazole treated patients compared to fluconazole treated patients with osseous coccidioidomycosis (118). Overall, they found itraconazole tended to be slightly more efficacious with fewer relapses compared to fluconazole treated patients. Studies using a murine model of CM demonstrated prolonged survival of mice infected with *Coccidioides* treated with either 50 mg/kg of itraconazole or fluconazole (125). At this same dose, they found equal clearing of fungi from both brain and kidney; however, itraconazole demonstrated an enhanced clearing of fungi in spinal cord and lungs.

#### Voriconazole

Voriconazole is often used for patients who are intolerant or refractory to other triazoles in the treatments of coccidioidomycosis (128, 129). The advantages of this antifungal agent include the availability of in both intravenous and oral formulations, high oral bioavailability, wide distribution throughout body, and the ability to penetrate the CSF (Table 1) (100). Nevertheless, voriconazole exhibits many attributes necessitating a working knowledge of its differences compared to other agents. Voriconazole possesses a variable half-life (patient dependent), many drug-drug interactions, hepatotoxicity, visual disturbances, rashes, alopecia, xerosis, and long-term toxicity concerns including cutaneous malignancy (129–134). Due to the variable half-life and the contraindication in patients with renal dysfunction, therapeutic drug monitoring is highly recommended (135).

The efficacy of voriconazole in the treatment of coccidioidomycosis has been demonstrated in retrospective series with favorable outcomes observed in the majority of reported cases including those with bone meningeal and non-meningeal disease (129, 130).

#### Posaconazole

Posaconazole was initially available only as an oral solution; however, bioavailability was a problem (136). Currently, an intravenous formulation and delayed release oral tablet are now available and the latter demonstrates significant improvement of absorption (137). Posaconazole has been shown to penetrate most sites of the body, but exhibits poor CSF penetration (138, 139). Common adverse effects caused by posaconazole treatment include gastrointestinal distress, hypokalemia, hypertension, peripheral edema, dry mouth, and headache (140, 141). Additionally, there are concerns of potential toxicity with high posaconazole concentrations (142); therefore, therapeutic drug monitoring is suggested (Table 1) (143).

Studies have shown the efficacy of posaconazole for the treatment of coccidioidomycosis in murine models (144, 145). One study demonstrated that mice treated with 10 mg of posaconazole showed >70% sterilization in the spleens and livers of *Coccidioides* infected mice while itraconazole treated mice resulted in no sterilization in the same tissues tested (144). Clinically, posaconazole treatment has shown efficiency in the treatment of refractory cases of coccidioidomycosis (129, 140, 146, 147).

#### Isavuconazole

Isavuconazole exist as a prodrug, isavuconazonium sulfate, which is cleaved by plasma esterases into the active moiety. This novel triazole is available in both oral and IV formulations, has a prolonged half-life (~130 h), high bioavailability, and is widely distributed through-out the body (Table 1). Additionally, isavuconazole has shown efficacy clinically against multiple disparate fungal pathogens including the endemic mycoses (148–152). Isavuconazole has been shown to cause adverse effects; the most commonly observed include gastrointestinal disorders (diarrhea and nausea/vomiting) and hypokalaemia (149).

Thus far, there is limited clinical data for the use of isavuconazole therapy on patients with coccidioidomycosis. A prospective study has demonstrated efficacy in the treatment of primary infection with *Coccidioides* (151) and a retrospective study has demonstrated the potential use of isavuconazole in coccidioidal meningitis in the salvage setting (153).

### Combination Therapy

It stands to reason that targeting multiple pathways using a combination of drugs would improve efficacy. However, clinical trials are lacking in the case of combination therapy against coccidioidomycosis. Interestingly, studies using a murine model of coccidioidomycosis have demonstrated the synergistic effects of combination therapy with caspofungin and amphotericin B deoxycholate increasing survival and decreasing fungal burden of mice compared to monotherapy with either treatment (154). This is noteworthy as the echinocandins have little activity against *Coccidioides* species and should not be used as monotherapy or outside of the salvage setting. Additional reports on the potential utility of combination therapy against coccidioidomycosis is scant, and includes murine models of infection and a single case reports/case series in the salvage setting (111, 155–157). Overall, these cases demonstrate the potential promise of the use of combination therapy against refractory coccidioidomycosis.

### New Drug Development

Although recent development of new and less toxic triazoles have been a welcome advance, there is a clear need for more effective and less toxic antifungal agents/therapies, particularly fungicidal oral agents. There are numerous agents currently in development with new modes of action and potentially reduced toxicity. A new formulation of itraconazole (SUBA-itraconazole) (158) has recently become available and clinical studies are ongoing. Novel amphotericin B formulations are currently in development (159). Additionally, some of the drugs in development exhibit broad-spectrum activity against multiple mycoses. Olorofim (formerly F901318) is an orotomide (inhibitor of dihydroorotate dehydrogenase) and has shown excellent *in vitro* activity against a number of fungal pathogens including *Coccidioides*, and murine models have suggested fungicidal activity (160) with a phase II clinical trial currently ongoing. Fosmanogepix (formerly APX001), a GPI-anchor inhibitor, has shown activity against a broad spectrum of fungal pathogens (161–166). A recent study evaluated the activity of prodrug APX001 and prodrug analogs against *C. immitis* and treatment with APX001 in *Coccidioides* infected mice resulted in significantly longer survival rates and reduced fungal burden than fluconazole or control treated mice (167). Another potential new drug, nikkomycin Z, a chitin synthase inhibitor, is nearing phase 2 clinical trials (168) and has shown similar promise in murine models of infection (169). Also in development are new glucan synthase inhibitors [rezafungin and ibrexafungerp (formerly SCY-078)] (170), a fungal mitochondrial inhibitor (T2307), and a histone deacetylase inhibitor (MGCD290), some with an unknown mode of action (ASP2397), and some repurposed from cancer therapy (sertraline and auranofin) (100, 159, 171, 172).

## Conclusion

**Figure 2:**
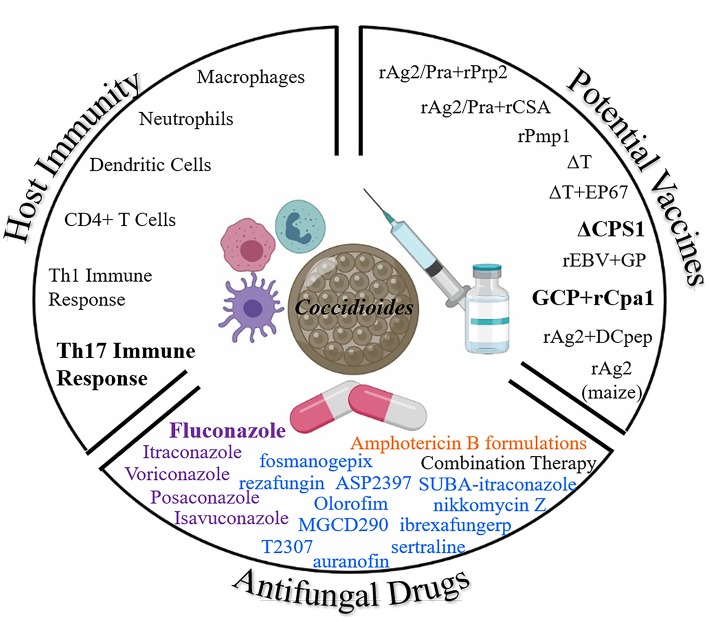
Three Arsenals to Combat *Coccidioides*. Here we highlight the current battle against *Coccidioides* from antifungals, potential vaccines, and the protective host immune response. Bolded terms: important for host protection, most common antifungal drug, and most promising current vaccine candidates against coccidioidomycosis. Color coding for antifungal drug classes: purple, Azoles; blue, drugs in development; and orange, Polyenes (Illustration created with BioRender).

Due to the rise of *Coccidioides* infections and concerns regarding toxicity of current antifungals, further research is needed to understand the protective host immune response, new less toxic antifungal drugs, and development of an effective vaccine to prevent coccidioidomycosis. Figure 2 demonstrates each of the three arsenals discussed in this paper in the battle against *Coccidioides*: host immunity, vaccines, and antifungal drugs. A prophylactic anti-*Coccidioides* vaccine would help to reduce cost associated with long term medical care and frequently needed life-long antifungal drugs. Live attenuated strains have been useful to elucidate our understanding of the protective host immune response against *Coccidioides* which requires T cell mediated immunity, particularly a Th1 and Th17 response. Novel formulations of adjuvants/delivery systems along with immunogenic *Coccidioides* antigens have also been discovered as vaccine candidates. Either could potentially be developed for clinical use. While fluconazole is currently the main antifungal of choice to battle coccidioidomycosis, studies are underway to find less toxic and effective drugs. Altogether, there remains a battle at hand to combat *Coccidioides*, the Dust Devil.

## Author Contributions

MV, GT, JG, and BB contributed to the writing, editing, and revision of the manuscript.

### Conflict of Interest Statement

JG is Chairman of the Board and a significant stockholder of Valley Fever Solutions, a company developing nikkomycin Z for the treatment of Valley fever. The remaining authors declare that the research was conducted in the absence of any commercial or financial relationships that could be construed as a potential conflict of interest.
